# Predictors and Modulators of Synthetic Lethality: An Update on PARP Inhibitors and Personalized Medicine

**DOI:** 10.1155/2016/2346585

**Published:** 2016-08-24

**Authors:** Stephen Murata, Catherine Zhang, Nathan Finch, Kevin Zhang, Loredana Campo, Eun-Kyoung Breuer

**Affiliations:** ^1^Department of Radiation Oncology, Stritch School of Medicine, Loyola University Chicago, Maywood, IL 60153, USA; ^2^Department of Otorhinolaryngology, University of Pennsylvania School of Medicine, Philadelphia, PA 19104, USA

## Abstract

Poly(ADP-ribose) polymerase (PARP) inhibitors have proven to be successful agents in inducing synthetic lethality in several malignancies. Several PARP inhibitors have reached clinical trial testing for treatment in different cancers, and, recently, Olaparib (AZD2281) has gained both United States Food and Drug Administration (USFDA) and the European Commission (EC) approval for use in* BRCA*-mutated advanced ovarian cancer treatment. The need to identify biomarkers, their interactions in DNA damage repair pathways, and their potential utility in identifying patients who are candidates for PARP inhibitor treatment is well recognized. In this review, we detail many of the biomarkers that have been investigated for their ability to predict both PARP inhibitor sensitivity and resistance in preclinical studies as well as the results of several clinical trials that have tested the safety and efficacy of different PARP inhibitor agents in* BRCA* and non-*BRCA*-mutated cancers.

## 1. Introduction

DNA damage can be acquired through endogenous and exogenous sources that, if left unrepaired, can contribute to genomic instability and oncogenesis. Indeed, defects in the DDR signaling pathway are often found in various human cancers [1–3]. The concept of “synthetic lethality” between two genes becomes relevant when a mutation to either separately is still compatible with viability, but mutations to both genes lead to death [4]. If an oncogenetic gene mutation is viewed as the first “hit,” targeting a partner gene or gene product should theoretically induce synthetic lethality in neoplastic cells. This therapy would also have minimal side effects on healthy cells with normal gene function [5]. A relevant example of synthetic lethality quickly moving to clinical application is the use of poly(ADP-ribose) polymerase (PARP) inhibitors for the treatment of* BRCA-*associated cancers.* BRCA1* and* BRCA2* are tumor suppressor genes encoding proteins that play important roles for DNA double-stranded break (DSB) detection for the homologous recombination (HR) repair pathway [6, 7]. Deficiencies in* BRCA1/2* function are associated with compromised HR repair, genomic instability, and oncogenesis [8–10]. PARP is a nuclear protein in the base excision repair (BER) pathway that recruits BER machinery to DNA single-stranded break (SSB) sites [5]. Inhibition of PARP would cause a collapse in the BER pathway and result in the accumulation of SSBs that break down to DSBs upon undergoing DNA replication [11–13]. In healthy cells, PARP inhibition would be of no large consequence because of effective DSB repair. However, in the context of* BRCA-*mutated cancers with compromised HR repair, breakdown of the BER pathway brought on by PARP inhibition would kill tumor cells from the buildup of DSBs [13–15]. The efforts to take advantage of synthetic lethality with PARP inhibitors have led to drug development for the treatment of patients with germline mutations in* BRCA1/2*. Olaparib is a PARP1/2 inhibitor that has gained approval by both the FDA and EC for use in patients with* BRCA*-mutated advanced, recurrent, platinum-sensitive serous ovarian cancer [16]. Meanwhile, Veliparib, Niraparib, Rucaparib, CEP9722, and BMN673 are all undergoing clinical trials to oversee their potential for treating common* BRCA*-associated cancers (Table 1) [17–21]. Several other PARP inhibitors that are mentioned in this review are being used in* in vitro* studies but have not yet been tested clinically.

In spite of the push to develop PARP inhibitors, opportunities for their optimal application remain largely unclarified. While defects in HR pathways signify opportunities for synthetic lethality, there is a push to look beyond* BRCA* mutational status to assess HR dysfunction, especially since only 15% of ovarian epithelial cancers are deficient in HR due to mutations of* BRCA1/2* [22, 23] and only 5–10% of breast and ovarian cancers are associated with* BRCA* germline mutation [24]. Meanwhile, it is increasingly apparent that HR defects are not always predicted by germline* BRCA* status. For example, several phase II clinical trials that stratified patients according to* BRCA1/2* germline mutational status showed less than 50% objective response rate (ORR) to Olaparib compared to control [25, 26]. It appears that a significant subset of sporadic cancers with “BRCAness,” a* BRCA-*like phenotype resulting from HR deficiencies, are also hypersensitive to PARP inhibitors. For example, Gelmon et al. showed that a significant fraction of ovarian and breast cancer patients with an intact* BRCA* gene responded to PARP inhibitors [27]. However, this expanded arsenal for PARP inhibitor therapy will remain untapped unless effective strategies are in place for patient stratification. Given the fact that BRCAness is a prerequisite for hypersensitivity to PARP inhibitors, the optimization of synthetic lethality relies on having biomarkers to predict BRCAness.

In this review, we detail select predictive and modulatory biomarkers for PARP inhibitors of clinical-translational significance that will help reap the benefits of personalized cancer therapy.

## 2. Biomarkers in the HR Pathway

### 2.1. Partner and Localizer of BRCA2 (PALB2)

PALB2 is a tumor suppressor [28] and binding partner of BRCA2 that facilitates the nuclear localization and HR capabilities of BRCA2 [28]. During HR, PALB2 association with RAD51 and DNA stimulates strand invasion [28]. Mutations in* PALB2* have been demonstrated in 1.1% of patients with familial breast cancer [29] and the c.1592delT frameshift mutation has been linked to a 6-fold increase in likelihood of developing breast cancer [30].* PALB2* mutations were also identified in 0.6% of patients with familial pancreatic cancer [31].* PALB2*-deficient lymphoblasts EUFA1341 cells displayed increased cytotoxicity in response to Olaparib compared to their controls [28]. Due to the fact that PALB2 helps to regulate BRCA2/RAD51-mediated HR and has demonstrated its ability to induce synthetic lethality in the presence of PARP inhibition, PALB2 deficiency in tumors is an interesting prospect for future clinical trials regarding PARP inhibitor sensitivity.

### 2.2. Fanconi Anemia (FA) Complementation Group (FANC)

FANC members include FANCD1 (BRCA2), FANCD2, FNAC31, and FANCN and play a major role in HR [32]. These proteins are related by their common association in a nuclear complex. After DNA damage, activation of the FA repair pathway involves the colocalization of FANCD2 with BRCA1 [33] in a manner dependent on monoubiquitination [34]. Thus, the functional biomarker of the FA pathway activation is nuclear FA protein/BRCA foci formation. The impairment of nuclear FA protein/BRCA foci formation after DNA damage is a powerful method for assessing functionality of the FA repair pathway [32, 35] and an important biomarker for HR defects. Powerful metrics are available to detect FANCD2/BRCA1 foci formation, such as the FA triple-staining immunofluorescence based method (FATSI), which identified a subset of non-small-cell lung cancer (NSCLC) tumors that were deficient in FANCD2/BRCA1 foci and thus were repair deficient [32]. Subsequently, these NSCLC cells were hypersensitive to Veliparib, BMN673, and ABT263 [32]. It was also shown that HeLa cervical cancer cells with defective FANCD2, FANCA, or FANCC exhibited cellular hypersensitivity to KU0058948 [4]. Thus, deficient FANCD2 manifested by absent foci formation after DNA damage may be a valuable biomarker to predict PARP inhibitor sensitivity.

### 2.3. Rad51

Rad51 is crucial for repair of DSBs via the HR pathway. RAD51 nucleates on single-stranded DNA molecules (ssDNA), which initiates the search for its homologous sequence and strand invasion [32, 36]. RAD51 also interacts with PALB2, BRCA1, and BRCA2 during HR [37, 38]. Formation of RAD51 nuclear foci in response to DNA damage is a functional biomarker for intact HR [39, 40] and lack of foci predicts deficient HR and breast cancer sensitivity to chemotherapy [35, 41]. Graeser et al. showed that sporadic breast cancers with lower Rad51 scores (Rad51 foci formation following anthracycline-based chemotherapy) showed decreased HR and increased sensitivity to anthracycline-based chemotherapy [42]. Furthermore, Rad51 paralog C deficiency caused Olaparib sensitivity in a gastric cancer xenograft model [43]. Increased sensitivity to KU0058948 was also observed in HeLa cells with deficient Rad51 and Rad54 [4]. Mukhopadhyay et al. demonstrated that 93% of ovarian cancer cells that showed no increase in Rad51 foci upon exposure to Rucaparib, and thus had deficient HR, subsequently showed cytotoxicity. Conversely, ovarian cancer cells that showed increased Rad51 foci, and thus had adequate HR, did not demonstrate cytotoxicity [44]. These results show that lack of Rad51 foci in response to DNA damage is a predictor of defective HR and thus can predict sensitivity to PARP inhibition.

## 3. Biomarkers in the DDR Pathways

### 3.1. Ataxia Telangiectasia Mutated (ATM)

ATM is autophosphorylated on Ser1981 in response to DNA DSBs and phosphorylates several proteins within the nucleus of mitotic cells, including BRCA1, p53, CHK2, RAD17, and RAD9, resulting in DSB repair and arrest of the cell cycle [45]. McCabe et al. showed that HeLa cells treated with an ATM kinase inhibitor or siRNA targeting ATM were hypersensitive to KU0058948 [4]. Furthermore, Williamson et al. showed that Granta519 and UPN2 mantle cell lymphoma cells with low ATM expression levels were hypersensitive to Olaparib compared to their controls [46]. Interestingly,* ATM* deficiency predicted PARP1 inhibitor sensitivity in p53-null gastric cancer cells, and it was speculated that combined inhibition of ATM and PARP1 is a potential therapy for* p53*-disrupted gastric cancer [47].

### 3.2. Serine-Threonine Protein Phosphatase (PP2A)

PP2A is a phosphatase in the Ser/Thr protein family with 4 regulatory subunits, PPP2R2A, PPP2R2D, PPP2R5A, and PPP2R3C. It is vital to DSB repair and activation of cell cycle checkpoints due to DNA damage [48] but has also been shown to negatively regulate ATM, CHK1/2, and other proteins necessary for DSB repair [48]. This may be explained by the fact that different PP2A complexes have different functions at different points of the repair process [48]. PPP2R2A dephosphorylates ATM at S367, S1893, and S1981, which mediates its retention at sites of DSBs and facilitates HR [48]. Kalev et al. showed that 40% of NSCLCs exhibited decreased PPP2R2A levels and consequently had increased phosphorylation of ATM at S1981, decreased retention at sites of DSBs, and decreased HR [48]. Also, HeLa cells treated with shRNAs specific for PPP2R2A and lung carcinoma cell lines with intrinsically decreased levels of PPP2R2A showed increased sensitivity to Veliparib in comparison to their respective controls [48]. These facts demonstrate the importance of PPP2R2A in maintaining ATM function integrity and the potential usage of decreased PPP2R2A expression as a predictor of PARP inhibitor sensitivity.

### 3.3. Mre11

Mre11 is part of the Mre11-Rad50-Nbs1 (MRN) complex, which contributes to DSB sensing and scaffolding of HR effector proteins at DSB sites [49]. Deficiency in* MRE11* is commonly found in endometrial cancer, and Koppensteiner et al. found that these* MRE11*-deficient endometrial cancers are hypersensitive to BMN673 [50]. Loss of Mre11 in head and neck cancer cells confers hypersensitivity to GPI15427 both* in vitro* and* in vivo* using a mouse xenograft [51]. Furthermore, Cal51 breast cancer cells [50] and various acute myeloid leukemia (AML) cell lines [52] with deficient Mre11 showed hypersensitivity to KU58948 and BMN673, respectively.

### 3.4. Tumor Protein p53 (TP53)

TP53 is a tumor suppressor in the DDR pathway that causes transient cell cycle arrest, senescence, and apoptosis in response to DNA damage [53]. Almost all* BRCA1*-mutated breast cancers have a deleterious* TP5*3 mutation, resulting from genomic instability-mediated complex and truncating mutations [54]. This suggests that* TP53* deficiency may represent a biomarker for BRCAness and hypersensitivity to PARP inhibitors [54, 55]. Furthermore, over 90% of basal-like breast cancers (triple-negative, high-grade breast carcinomas) have a deleterious* TP53* mutation and exhibit a molecular phenotype reminiscent of* BRCA1*-deficient breast cancer [54]. A recent study showed that depletion of TP53 in various breast cancer cell lines displayed hypersensitivity to the PARP inhibitor IQD in comparison to their respective controls [56].

### 3.5. *γ*H2AX

*γ*H2AX is a variant of the H2A histone family that is phosphorylated on Ser139 by ATM and ATM-Rad3-related (ATR) in the PI3K pathway of DNA repair and functions to recruit other DNA repair proteins in response to DNA damage [57, 58]. Importantly, *γ*H2AX foci form in response to DSBs [57], and the presence of foci can be utilized as a biomarker to measure DNA damage induced by PARP inhibition [59].* BRCA1*-mutated acute myeloid leukemia cells that were exposed to Olaparib subsequently formed *γ*H2AX foci, suggesting that *γ*H2AX foci formation may be a useful biomarker for successful PARP inhibition [59]. Furthermore, there have been two completed phase I trials for Veliparib in which investigators found that *γ*H2AX was a reliable biomarker to measure sensitivity to PARP inhibition of circulating tumor cells of metastatic solid tumors or lymphomas [60, 61].

## 4. Biomarkers in the BER Pathway

### 4.1. Poly(ADP-Ribose) (PAR)

PAR chains are linear and branched chains of up to 200 ADP-ribose units whose formation is catalyzed by PARP1/2 [62, 63]. PARPs play a significant role in BER, and PARylation acts as a specific indicator of PARP activity in DNA repair. PARylation also plays a role in chromatin modification, transcription, telomere cohesion, cell death, insulator function, mitotic apparatus function, and energy metabolism [62, 64], which affect genome stability, inflammation, neuronal function, aging, and carcinogenesis [64]. A subset of head and neck cancers has an elevation in basal PARylation [65]. Interestingly, head and neck cancer cells with elevated PAR are hypersensitive to Veliparib [65], suggesting that high PAR levels predict sensitivity to PARP inhibition. Further studies should be done to determine the significance of elevated PAR in different tumors.

### 4.2. PARP1-Binding Protein (PARP-BP)

PARP1-binding protein (PARP-BP) is encoded by the gene C12orf48 and directly interacts with PARP1 to enhance its activity and the repair of DNA breaks [66]. Its expression is upregulated in pancreatic ductal adenocarcinomas (PDACs) and a number of other malignancies, which indicates increased PARP activity. Knockdown of C12orf48 in PDACs decreased PARP-BP expression, which subsequently caused decreased PARP1 activity and cell viability, while increasing sensitivity to Adriamycin, UV irradiation, and hydrogen peroxide [66]. This highlights the importance of PARP1 activity in the viability of PDACs with upregulated PARP-BP and should be further explored to determine if these tumors may be hypersensitive to PARP inhibitors.

### 4.3. X-Ray Repair Cross-Complementing 1 (XRCC1)

XRCC1 is a key player in the DNA BER pathway, which is recruited in response to PAR chain formation at SSB sites by PARP1 [67].* XRCC1* is deficient in 16% of breast cancers and is associated with high grade, triple negativity, loss of hormone receptors, and basal-like breast cancers [68].* XRCC1-*deficient Chinese hamster ovary (CHO) EM9 cells showed accumulation of SSBs [67] and were hypersensitive to PARP inhibitors due to the supplementary effect of PARP inhibitors in preventing DNA ligation [67]. Mouse fibroblasts deficient in* XRCC1* were hypersensitive to the PARP inhibitor 4-amino-1,8-naphthalimide (4-AN) [69], and XRCC1 knockdown breast cancer cells were hypersensitive to KU0058948 [70].

## 5. “Other” Biomarkers

Lastly, there are other proteins and abnormalities in DNA expression that do not play a direct role in HR or DDR but can indirectly affect the process. These “other biomarkers” indirectly affect DNA repair through regulation of BRCA1/2, ATM, or other proteins responsible for its execution. Thus, their abnormal expression may be predictive biomarker for PARP inhibitor sensitivity.

### 5.1. E26 Transformation Specific or E-Twenty-Six (ETS)

*ETS* genes belong to a large family of transcription factors that regulate cell differentiation, proliferation, migration, cell cycle control, apoptosis, invasion, and angiogenesis [71, 72].* ETS* gene fusions occur widely in many cancers including Ewing's sarcoma, acute myeloid leukemia (AML), and prostate cancer [73]. Baker et al. found that ETS-2 complexes with components of SWI/SNF repress* BRCA1* in MCF7 cells [74]. ETS-1 expression is a poor prognostic marker for breast, lung, colorectal, and ovarian cancer [71, 75, 76]. Interestingly, Legrand et al. revealed that ETS-1 activates the catalytic activity of PARP1, which then PARylates ETS-1 [76], revealing a novel link between ETS-1 and DDR pathways. They also found that PARP inhibition upregulates* ETS-1* transcriptional activity and led to its nuclear accumulation and selective cytotoxicity in ETS-1 expressing HeLa cells [76]. This suggests that nuclear ETS-1 expression may be a predictive biomarker for PARP inhibitor sensitivity. However, in a phase 1 dose-escalation study, no correlation was found between* ETS* gene rearrangement and sensitivity to Niraparib in prostate cancer [20]. Further studies should be performed to determine the significance of nuclear ETS-1 expression in PARP inhibitor sensitivity.

### 5.2. Transforming Growth Factor *β* (TGF*β*)

TGF*β* is a cytokine whose presence at tumor sites has classically been associated with poor prognosis [77]. TGF*β* has been shown to inhibit the expression of ATM, mutS homolog 2 (MSH2), and BRCA1 in BT474 breast cancer cells through microRNA, specifically the miR-181 family [77], inducing a BRCAness phenotype. Similarly, treatment of MDA-MB-231, MDA-MB-468, and BT474 breast cancer cells with TGF*β* caused increased sensitivity to Veliparib [77]. Thus, the presence of increased TGF*β* signaling may be an indicator of BRCAness and subsequent hypersensitivity to PARP inhibition.

### 5.3. MicroRNAs (miRNAs)

miRNAs are small noncoding RNAs that mediate posttranscriptional repression and degradation of mRNA transcripts [77, 78]. Usage of miRNA is an effective and clinically tolerable method for inducing BRCAness and hypersensitivity to PARP inhibitors. The miR-181 family is induced by TGF*β* to suppress ATM, MSH2, and BRCA, promoting BRCAness, as previously described [77]. Furthermore, more aggressive breast cancers exhibited increased expression of miR-181 [79]. Similarly, miR-182 downregulates* BRCA1* expression in various breast cancer cell lines* in vitro* and* in vivo*, resulting in defective HR-mediated repair and increased sensitivity to irradiation and Olaparib [80]. Mouse xenograft of MDA-MB-231 cells stably expressing miR-182 showed increased PARP inhibitor sensitivity to 4-amino-1,8-naphalamide (ANI) and Veliparib compared to their controls [80]. miR-103 and miR-107 target Rad51 and inhibit formation of Rad51 foci in response to DNA damage in osteosarcoma cells [81, 82] and subsequently increase sensitivity to Olaparib [81]. Furthermore, it was demonstrated that ovarian cancer cells with high levels of hsa-miR-107 were sensitive to Olaparib, and inhibition of hsa-miR-107 eliminated this sensitivity. Similarly, overexpression of miR-96 in osteosarcoma U2OS cells reduced the levels of Rad51 by directly targeting its coding region [83], decreasing the efficiency of HR and enhancing sensitivity to Olaparib.

### 5.4. Lysine-Specific Demethylase 1 (LSD1)

LSD1 is an epigenetic regulator of gene expression that demethylates histones H3K4 and H3K9 [84]. LSD1 regulates genes associated with proliferation including those for p21, ErbB2, and Cyclin A2 [84]. LSD1 is upregulated in many cancers and is a predictive biomarker for aggressive biology in breast cancer and prostate cancer [84]. Increased LSD1 levels show a positive correlation with progression, proliferation, and invasion of breast cancer cells [85], and pharmacological inhibition of LSD1 results in growth inhibition [84]. One study found that LSD1 is recruited to sites of DNA damage in a manner dependent on Ring Finger Protein 168 (RNF168), suggesting its potential role in DDR downstream of RNF168 [86]. Interestingly, ectopic expression of LSD1 in basal-like breast cancer cells promoted downregulation of BRCA1 and hypersensitivity to Olaparib [87]. Thus, because LSD1 is upregulated in various cancers, including breast and prostate, and there is preliminary evidence of LSD1-dependent BRCA1 suppression and PARP inhibitor sensitivity, further clinical validation is required to explore LSD1 as an official biomarker for responsiveness to PARP inhibitor.

### 5.5. Cyclin-Dependent Kinase 12 (CDK12)

CDK12 is a kinase that regulates cell cycle checkpoints and positively regulates BRCA1 [88]. It is mutated in nearly 3% of high-grade serous ovarian cancers, resulting in reduced BRCA1 levels and compromised HR repair. CDK12 is also a key regulator in the transcription of several other genes involved in DNA repair including ATM/ATR, FANCL, and BRCA2 [89]. As such, tumors with mutated* CDK12* should show “BRCAness” and are candidates for PARP inhibitor therapy [90]. A study has shown that* CDK12*-deficient ovarian cancer cells are more sensitive to Veliparib compared to their controls [90]. Furthermore, serous ovarian carcinoma cells with mutated* CDK12* exhibit hypersensitivity to Olaparib [91], platinum derivatives, and alkylating agents [89]. This provides evidence that* CDK12*-deficient ovarian cancers could be targets for PARP inhibitor therapy, and further work should be done to evaluate PARP inhibitor efficacy in other CDK12-deficient tumors.

### 5.6. Transforming Acidic Coiled-Coil Containing Protein 3 (TACC3)

TACC3 is a member of the TACC family, which consists of proteins that localize at centrosomes to facilitate microtubule assembly and stabilization, enabling chromosomal integrity during mitosis [92]. TACC3 has been shown to regulate microtubule nucleation by interacting with *γ*-tubulin ring complex proteins [93] and promoting plus-end microtubule growth [94]. Both upregulation and downregulation of TACC3 are found in human solid tumors [95, 96]. Silencing of TACC3 results in microtubule destabilization and chromosome misalignment [92]. Overexpression of TACC3 has been shown to lead to accumulation of DSBs and negative regulation of* ATM* and subsequent DDR signaling in U2OS cells [97]. Overexpression of TACC3 also impairs HR, NHEJ, and normal cell cycle checkpoint function in U2OS cells [97]. Moreover, it was found that nontumorigenic human mammary epithelial MCF10A cells with elevated levels of TACC3 showed hypersensitivity to Olaparib and NU1025 [97], suggesting its potential role in conferring synthetic lethality. Further studies with different cell lines should be employed to determine the role of TACC3 overexpression in conferring hypersensitivity to PARP inhibition.

### 5.7. Aurora Kinase A (Aur A)

Aur A is a protein kinase necessary for construction of the mitotic spindle [98] and phosphorylates cell division cycle 25 homolog B (CDC25B) at the G2/M checkpoint, causing CDK1 activation and mitotic entry [99]. Cazales et al. showed that, during DNA damage-induced activation of the G2/M checkpoint, Aur A was not activated, and the cell cycle did not progress in U2OS cells [99]. However, ectopic expression of activated Aur A resulted in a bypass of this checkpoint [99]. Aur A is overexpressed in various solid tumors, including ovarian cancer [100], cervical cancer [101], and colon cancer [102]. Sourisseau et al. demonstrated that overexpression of Aur A impairs formation of Rad51 foci in MCF10A cells and HR in human embryonic kidney 293 (HEK293) cells. Overexpression of Aur A in PIR12 (Capan 1-derived PARP inhibitor resistant cell line) also induced sensitivity to KU0058948 [98]. Taken together, these data suggest that Aur A overexpression may predict sensitivity to PARP inhibition.

### 5.8. Phosphatase and Tensin Homolog (PTEN)

PTEN is a tumor suppressor that inactivates the P13K/AKT pathway whose signaling is important for propagation of the cell cycle [103].* PTEN* deficiency is associated with many malignancies, including breast [104] and prostate cancers [105], and disrupts chromosomal integrity by causing centromere breakage and translocations [106]. Mendes-Pereira et al. showed that* PTEN* deficiency leads to impairment of HR, which subsequently leads to increased sensitivity to KU0058948 in HCT116 colorectal carcinoma and HEC1A endometrial adenocarcinoma cells [107]. There are numerous reports of* PTEN*-deficient cancer cell lines that show decreased Rad51 levels [106, 107] and increased nuclear H2AX foci [106, 108, 109], suggesting deficient HR that leads to accumulation of foci. Furthermore, Shen et al. showed that PTEN potentiates activation of the* Rad51* promoter by E2F-1 in PC3 prostate cancer cells [106]. However, Fraser et al. showed that* PTEN-*deficient prostate cancer cells do not have decreased Rad51, have sufficient HR, and are insensitive to PARP inhibitors [108, 110]. This discrepancy may be explained by the fact that prostate cancer cells with an intrinsically null* PTEN* genotype have other genomic aberrations that are not present in prostate cancer cells with experimentally silenced PTEN, which can cause different behaviors [111]. Regardless, further studies should be pursued to examine the role of PTEN in HR in prostate cancer to determine its utility as a biomarker for predicting PARP inhibitor sensitivity.

### 5.9. Mitochondrial DNA (mtDNA)

There is emerging evidence linking mtDNA depletion with BRCA2 depletion. mtDNA is depleted in breast, prostate, and thyroid transformed cells [112], which promotes activation of calcineurin/PI3Kinase/AKT signaling that causes upregulation of miR-1245 and ubiquitin ligase Skp2, negative regulators of BRCA2 [112]. This promoted HR deficiency and increased sensitivity to Rucaparib [112]. Further studies with a larger sample size are needed to further validate this promising correlation and to determine whether mtDNA depletion can be used as a biomarker for PARP inhibitor sensitivity.

### 5.10. Genomic Scar

A genomic scar is defined as a genomic abnormality [113] that is present in a wide variety of cancers, including breast, ovarian, pancreatic, esophagus, lung, and prostate cancers [113]. Several known genomic scars have been associated with BRCA1/2 dysfunction and homologous repair dysfunction [113]. Telomeric allele imbalance (*N*_tAi_), loss of heterozygosity (LOH) clustering, mutational signature 3 (mutational signature D), and total number of somatic, synonymous, and nonsynonymous coding mutations (*N*_mut_) are genomic scars that are predictive of BRCA1/2 dysfunction [113] and thus may predict responsiveness to PARP inhibitors. Homologous recombination defects (HRD) and large-scale transitions are genomic scars predictive of general HR dysfunction and may also indicate responsiveness to PARP inhibitors [113–116]. Further studies to evaluate the direct relationship between these genomic scars and sensitivity to PARP inhibitors should be performed.

## 6. Discussion

It is accepted that PARP inhibition mediates synthetic lethality in tumors with inherited BRCA deficiencies [117] and that BRCA1 hypermethylation can predict sensitivity to PARP inhibition [118–121]. However, it is increasingly clear that deficient* BRCA1/2* germline status is not enough to predict PARP inhibitor sensitivity [48, 122–124]. This growing sense is supported by clinical trials, which have shown that not all* BRCA1* mutation carriers are responsive to PARP inhibition [26, 125]. Altogether, these results further underline the need for nuanced biomarkers predictive of PARP inhibitor hypersensitivity. This entails a paradigm shift away from reliance on single predictive biomarkers for PARP inhibitor hypersensitivity (such as deficient* BRCA1* gene status) and towards the idea of predictive algorithms and biomarker codes that characterize various manifestations of “BRCAness” [126].

Additionally, we need to understand the roles of PARP1/2 outside of BER that contribute to the “off-target” effects of PARP inhibition, which induce cytotoxicity through mechanisms separate from dysfunctional HR repair, and can modulate or amplify the net synthetic lethal effect of PARP inhibitors. Even though synthetic lethality is mediated by PARP inhibition, not all benefits of PARP inhibitors are mediated through defects in HR repair. For example, while the canonical role of PARP is through the DNA damage repair pathway, PARP also plays a role in various networks including tumor-promoting inflammation, cell cycle checkpoint regulation, senescence, angiogenesis, epithelial-mesenchymal transition (EMT), PARylation and remodeling of chromatin during transcription, and programmed cell death and metastasis [14, 127–130]. These off-target mechanisms may modulate the tumor microenvironment through a scenario known as “contextual” synthetic lethality that further sensitizes tumor cells to PARP inhibition [131]. This concept is exemplified in the emerging link between PARP inhibition and suppression of angiogenesis [130, 132–134]. Interestingly, hypoxic conditions deregulate DNA repair pathways and promote genomic instability through deregulation of BRCA1/2 [135–137]. Meanwhile, it is also postulated that PARP inhibitors inhibit BER to cause transient stalling of replication forks that degenerate into DSBs [138, 139].

It is also important to consider the way PARP inhibition relates to nononcogenic addiction, which is defined as the hyperreliance on secondary regulatory pathways in response to stressful conditions brought on by oncogene activation and tumor progression [140, 141]. Interestingly, nononcogenic addiction may represent underlying HR defects that can be targeted through synthetic lethality. For example, elevated PAR levels indicate PARP hyperactivity in response to underlying HR defects [65, 142, 143]. Thus, high PAR levels may indicate compensatory dependence on the BER pathway and therefore hypersensitivity to PARP inhibition [144]. As such, awareness of nononcogenic addiction events may enable us to distinguish between biomarkers of primary HR deficiencies versus biomarkers of secondary compensatory events both of which may predict hypersensitivity to PARP inhibition.

While the contextual parameters of PARP inhibition may be leveraged to our advantage, this may be a double-edged sword due to the addition of a new layer of complexity to the development of predictive biomarkers. For example, increased TGF*β* signaling caused hypersensitivity to PARP inhibition in BT474 but not in MCF7 cells [145]. Meanwhile, knockdown of* ATM* by siRNA significantly increased sphere-forming efficiency (SFE) in BT474 and MDA361 but not in MCF7 cells. While these variations showcase the cell type-dependent outcomes of ATM regulation by TGF*β* [146], they also highlight the possibility that contextual variables may inflate the perceived efficacy of PARP inhibition. Awareness of such possibilities helps guard against falsely attributing net cytotoxic effects to a single biomarker, since the observed efficacy of PARP inhibition may really be the sum effect of multiple mechanisms, not necessarily all due to synthetic lethality due to HR defects.

It is crucial to proceed with caution when identifying candidate predictive biomarkers. One example is the upregulation of EMSY, a putative oncogene that transcriptionally silences exon 3 of* BRCA2* that links the BRCA2 pathway to sporadic breast and ovarian cancer [147]. It was suggested that because EMSY amplification could mimic a* BRCA2* mutated state [148], it could account for BRCAness in sporadic breast and ovarian cancers with intact* BRCA2* [122] and possibly predict hypersensitivity to PARP inhibitors. However, it was recently shown that cells with an amplified EMSY had the same RAD51 foci formation efficacy, as well as sensitivity to PARP inhibitors, as cells without EMSY amplification [149]. Taken together, these results underscore the importance of triangulating BRCAness through a variety of biomarkers in order to detect opportunities for synergism, avoid conflation of various cytotoxic mechanisms, and customize treatment.

## Figures and Tables

**Table 1:** PARP inhibitors currently undergoing development.

PARP inhibitors	Tumor types	Most advanced developmental stage in progress	Key clinical trials	References
Olaparib	FDA approved for ovarian cancer Being tested for breast, prostate, and pancreatic cancers	FDA approved	Phase II trial found that Olaparib monotherapy in patients with BRCA1/2 mutated ovarian cancer following ≥3 chemotherapy treatments resulted in 31% response rate Phase II trial found that Olaparib maintenance therapy in patients with platinum- (Pt-) sensitive recurrent serous ovarian cancer with mutated BRCA1/2 resulted in median progression free survival of 6.9 months longer than those receiving placebo	[150, 151]

Talazoparib	Being tested for ovarian, breast, and various advanced/metastatic solid cancers (primary peritoneal carcinoma, fallopian tube carcinoma, etc.)	Phase III	Phase II trial for Talazoparib monotherapy in patients with deleterious BRCA1/2 mutated ovarian cancer who had prior PARP inhibitor treatment currently recruiting Phase III trial for Talazoparib monotherapy in patients with BRCA1/2 mutated, advanced, or metastatic breast cancer currently recruiting	[152, 153]

Veliparib	Being tested for breast, pancreatic, non-small-cell lung cancers, lymphoma, and multiple myeloma, mostly in combination with chemotherapy	Phase III	Phase I/II trial of Veliparib and Topotecan for relapsed ovarian cancer of negative or unknown BRCA status completed Phase II trial of Veliparib alone or with Gemcitabine and Cisplatin in patients with locally advanced or metastatic pancreatic cancer currently recruiting Phase I/II trial for Veliparib, Bendamustine HCl, and Rituximab in patients with relapsed lymphoma and multiple myeloma completed	[154–156]

Rucaparib	Being tested for ovarian and pancreatic cancers	Phase III	Phase III trial for Rucaparib maintenance therapy in patients with Pt-sensitive recurrent ovarian cancer, fallopian tube, or primary peritoneal cancers currently recruiting Phase II trial for Rucaparib monotherapy in patients with BRCA1/2 mutated, locally advanced, or metastatic pancreatic cancer currently ongoing	[157, 158]

Niraparib	Being tested for ovarian and breast cancers and Ewing sarcoma	Phase III	Phase III trial for Niraparib monotherapy in patients with HER2 negative, BRCA1/2 mutated breast cancer currently recruiting Phase II trial for Niraparib monotherapy in patients with ovarian cancer following ≥3 chemotherapy treatments currently recruiting	[159–161]
